# Scholarship of Discovery and Beyond: Thinking About Multiple Forms of Scholarship and Elements of Project-Based Learning to Engage Undergraduates in Publishable Research

**DOI:** 10.3389/fpsyg.2019.00917

**Published:** 2019-04-24

**Authors:** Jeanine L. M. Skorinko

**Affiliations:** Department of Social Science and Policy Studies, Worcester Polytechnic Institute, Worcester, MA, United States

**Keywords:** multiple forms of scholarship, project-based learning (PBL), undergraduate, publication, diversity

Boyer (1990) encouraged academia to expand its definition of scholarship to include the scholarship of discovery, teaching, integration, and engagement. Since then, institutions of higher education have implemented mechanisms or policies that recognize multiple forms of scholarship (O'Meara, 2006). Proponents of this argue that it can help diversify and appropriately recognize faculty work (Park, 1996; Creamer, 1998), and create better alignment between faculty endeavors and institutional missions and goals (Diamond, 1999). Research indicates that implementing policies that encourage multiple forms of scholarship increases the likelihood that faculty: diversify the types of scholarship they engage in, feel satisfied, and want to stay at their institution (O'Meara, 2005). In addition, research shows that institutional effectiveness increases when polices include multiple forms of scholarship (O'Meara, 2006). At my own institution, a new promotion policy went into effect in 2017 that recognizes multiple forms of scholarship, and we received funding from the National Science Foundation to help with the advancement of women within this new policy (Skorinko et al., 2018). Therefore, I felt it was important in a special issue on engaging undergraduates in publishable research to consider how to work with undergraduates through multiple forms of scholarship.

One way in which I think that we can engage students in publishable work in each of the forms of scholarship is through project-based learning (Blumenfeld et al., 1991; Barron et al., 1998; Bell, 2010). In project-based learning, students participate in a project that examines a problem related to what they are learning. Project-based learning positively influences learning (Bell, 2010), and increases learning motivation, helps change student's thinking (Blumenfeld et al., 1991), and is considered a high impact practice (Kuh, 2008). Reflection on how the project relates to what is being learned is an important component of effective project-based learning (Barron et al., 1998). The key elements involved in setting up a project include: (1) setting appropriate learning goals, (2) developing authentic questions, (3) requiring sustained inquiry, (4) enabling students to take the driver's seat, and (5) encouraging reflection of how the project relates to their learning (Barron et al., 1998; Buck Institute for Education, 2018).

While there is work on problem-based learning (Dahlgren and Dahlgren, 2002; Hmelo-Silver, 2004) and service learning (Fleck et al., 2017) in psychology, there is less work directly examining project-based learning. While the literature may not market an approach as project-based learning, this type of learning is often hinted at in teaching of psychology practices. For instance, a recent paper suggested different ways to implement research into Introduction to Psychology courses to improve and unify the experience for undergraduates, but never calls any of the approaches project-based learning (Gurung and Hackathorn, 2018). Likewise, project-based learning is often used in undergraduate research methods courses, even if it is not referred to directly by name (Chapdelaine and Chapman, 1999; Pliske et al., 2015). At some institutions, fourth year students are encouraged (or required) to complete a research project (or senior thesis)—or engage in project-based learning. Thus, psychologists often engage in project-based learning, even if they do not directly refer to this technique by name.

By considering how to engage undergraduates into multiple forms of scholarship through a project-based learning framework, we can expand learning outcomes, diversify the work we do as psychologists, and encourage different forms of thinking amongst ourselves and our students. I will consider three forms of scholarship (discovery, teaching, and engagement) and how we can use project-based learning to engage students in publishable research.

## Scholarship of Discovery

The scholarship of discovery is the gold standard within Psychological Science. It involves engaging in research endeavors that expand our knowledge (Boyer, 1990). We may apply for research funding to support these endeavors, and we share our knowledge through conference presentations, peer-reviewed publications, book chapters, and books. Many of the articles in this special edition feature strategies for engaging students, including diverse students (Chan, 2019; Frohardt, 2019; Peifer, 2019; Ahmad et al., under review) in publishable work using the scholarship of discovery. As mentioned earlier, one mechanism for engaging undergraduates in publishable work within this type of scholarship is to use project-based learning in a research methods and/or statistics course to heighten their understanding of research and statistics (LoSchiavo, 2018; McKelvie and Standing, 2018; Mendoza and Martone, 2019).

In addition, many psychologists run research laboratories where they engaged students in research. Many articles in this special issue feature strategies that can be implemented to engage students in publishable research from a research lab perspective (Adams, 2019; Dunbar, 2019; Holmes and Roberts, 2019; Mendoza and Martone, 2019; Overman, 2019; Reavis and Thomas, 2019; Scisco et al., 2019; Stefanucci, 2019; Wood, 2019; Scherman, under review). Some researchers use collaborations to expand the possibilities of publishable work with undergraduate students (Bukach et al., 2019; Hammersley et al., 2019). Others engage students in publishable work through direct replication projects (Strand and Brown, 2019; Wagge et al., 2019). And, some engage undergraduates in cross-cultural research projects (Ashdown, 2019; Burns-Cusato and Cusato, 2019; Hill and Karlin, 2019).

In many cases, project-based learning is how students engage in a research methods course or in a research lab. However, it is important to consider the key elements of project-based learning (see above) and incorporate them into a sustained inquiry. Utilizing these elements can increase student learning, motivation, engagement, and the likelihood of publishable work. For instance, I incorporate reflection in the research lab. As a lab, we reflect through discussions or a written reflection on what we learned from the projects conducted. These reflections help students synthesize how the project they are working on connects to other concepts they are learning (design, methods, ethics, statistics, etc.), and the reflections enable me to figure out what is working and what needs tweaking.

## Scholarship of Teaching

The scholarship of teaching is also a natural fit for psychologists. In this form of scholarship, researchers investigate processes for teaching and learning effectively. It is argued that the scholarship of teaching “must be public, available for peer review and critique according to accepted standards, able to be reproduced and built on by other scholars” (Glassick, 2000, p. 879). One mechanism of making the work public is to publish it in teaching/learning journals within the field (or via special issues). For instance, in this special issue, we had one manuscript describe publishable research experiences from an undergraduate perspective (Matthews and Rose, 2018) and another provided perspectives from a faculty member and an undergraduate (Mendoza and Martone, 2019).

On the surface, it may seem more difficult to engage undergraduate students in this form of research (other than being participants). However, using a project-based learning framework, it becomes easier to see ways to engage students in this form of scholarship. For instance, one project that students could engage in is developing a teaching demonstration that highlights a key theory/component from that class. Those who develop creative and potentially effective demonstrations could then work with their professor to publish their demonstration in a teaching-related journal or other public venue (podcast, YouTube). This was a project in my Psychology of Gender course when I was an undergraduate. While my demonstration was not creative enough to move to the publication phase, other students' demonstrations have been (Ganske and Hebl, 2001; Hebl and King, 2004; Knight et al., 2004; Hebl et al., 2008; Fa-Kaji et al., 2016). These types of projects facilitate learning by requiring students to take a deep dive into a topic and clearly demonstrate why the topic is relevant to the course. Moreover, these projects/publications could be even more meaningful to undergraduates as they have something tangible to show—a demonstration. In addition, to publish this type of work, a sound methodology of determining its effectiveness on learning is required. This type of project could be integrated into any psychology course, such as a topic course like psychology of gender or human sexuality. It is particularly relevant to courses on learning, education, and teaching.

## Scholarship of Engagement

The scholarship of engagement is another avenue of scholarship for psychologists, especially those with applied interests. The scholarship of engagement involves the integration of theoretical and applied research and works with local, regional, national, or international communities. Some academic institutions have missions to work and give back to their local communities and one way of doing that is through engaged scholarship (Stanton, 2012). As with the scholarship of teaching, the scholarship of engagement needs to be publicly available and open for peer-review. Again, one mechanism of making the work public is through publication in an applied or community psychology journal (e.g., the Journal of Social Issues). Publication could also take other forms, such as an op-ed, amicus brief, creation/publication of an app or computer program, or the integration of some of the work/materials by a community organization.

Again, project-based learning provides a framework to engage undergraduates in this form of scholarship, enhance their learning, and increase the likelihood of producing something publishable. This could be a project in a course (see Smirles, 2011; Fleck et al., 2017), a project through a lab (see Schlehofer, 2018), or a project required in a curriculum (department or institution; see WPI, 2018). In my own experiences, I have utilized all three approaches. For instance, in Human Sexuality, students conduct a Public Service Announcement (PSA) project where they have the option of working with a local organization. The goal of this project is to pick a topic that is important to them and develop an effective public service announcement for that topic and relevant organization. While certainly not a traditional form of publication, the students work is public and organizations may benefit. Students have designed (and sent) PSAs to domestic violence shelters, the Capetown Holocaust and Genocide Center, Planned Parenthood, and several on-campus student organizations. This project enables students to synthesize what they learned and apply it to a real-world context.

In addition, I collaborate with colleagues in computer science and robotics engineering to develop assistive technologies for individuals with disabilities. We engaged undergraduate students in these projects whether on a volunteer basis or via their third or fourth year required projects. Currently, a colleague and I have a grant with the Disabled Persons Protection Commission in Massachusetts to develop assistive technologies for individuals with intellectual and developmental disabilities to recognize, report, and respond to abuse. We recruited interested undergraduate students (i.e., computer science and psychological science) to work on the project with us. These students will be conducting focus groups and interviews with individuals and their caregivers. They will also develop an app, computer program, or other assistive technology. Depending on their level of interest and engagement, they will assist with other forms of presentation and publication that we complete (e.g., op-eds, conference proceedings—a standard in computer science, or peer-reviewed publications). Working on this project, undergraduates are able to put theory into practice—an important learning outcome.

## Conclusion

While the engagement of undergraduates in the scholarship of discovery is the main focus for many psychologists, it is important to think about the multiple forms of scholarship and how we can engage ourselves, as well as undergraduates, into these forms of scholarship. Project-based learning in the classroom, research lab, or curriculum is a high impact learning practice. While it may start through instructions from a project leader, one aim is to help students foster their own insights, and then apply those insights to different research questions in the future. Project-based learning is also one way to engage students in publishable research regardless of the type of scholarship one engages in. By thinking more broadly about the types of scholarship we can engaged undergraduates in and the ways in which we can make that work public, we can increase learning outcomes for students, as well as the diversity of the faculty, the undergraduate students, and scholarship conducted within psychological science.

## Author Contributions

The author confirms being the sole contributor of this work and has approved it for publication.

### Conflict of Interest Statement

The author declares that the research was conducted in the absence of any commercial or financial relationships that could be construed as a potential conflict of interest.
